# Evaluation of Inflammation and Oxidative Stress Markers in Patients with Obstructive Sleep Apnea (OSA)

**DOI:** 10.3390/jcm12123935

**Published:** 2023-06-08

**Authors:** Maria Carmina Pau, Angelo Zinellu, Arduino A. Mangoni, Panagiotis Paliogiannis, Maria Roberta Lacana, Sara Solveig Fois, Sabrina Mellino, Alessandro G. Fois, Ciriaco Carru, Elisabetta Zinellu, Pietro Pirina

**Affiliations:** 1 Department of Medicine, Surgery and Pharmacy, University of Sassari, 07100 Sassari, Italy; 2 Department of Biomedical Sciences, University of Sassari, 07100 Sassari, Italy; 3 Discipline of Clinical Pharmacology, College of Medicine and Public Health, Flinders University, Bedford Park, SA 5042, Australia; 4 Department of Cliical Pharmacology, Flinders Medical Centre, Southern Adelaide Local Health Network, Bedford Park, SA 5042, Australia; 5 Clinical and Interventional Pulmonology, University Hospital of Sassari (AOU), 07100 Sassari, Italy; 6 Quality Control Unit, University Hospital of Sassari (AOU), 07100 Sassari, Italy

**Keywords:** obstructive sleep apnea, inflammation, oxidative stress, albumin, combined indices of inflammation

## Abstract

**Background:** The identification of circulating markers of oxidative stress and systemic inflammation might enhance risk stratification in obstructive sleep apnea (OSA). We investigated the association between specific haematological parameters, as easily measurable markers of oxidative stress and inflammation, and the degree of hypoxia during polysomnography using the apnea hypopnea index (AHI), oxygen desaturation index (ODI), and oxygen saturation (SpO_2_), in OSA patients. **Methods:** Associations between polysomnographic parameters and demographic, clinical, and laboratory characteristics were assessed in a consecutive series of patients with OSA attending the Respiratory Disease Unit of the University Hospital of Sassari, north Sardinia (Italy), between 2015 and 2019. **Results:** In 259 OSA patients (195 males and 64 females), the body mass index (BMI) was significantly and positively associated with the AHI and ODI, and negatively associated with the mean SpO_2_. No haematological parameter was independently associated with the AHI or ODI. By contrast, albumin, neutrophil, and monocyte counts, and the systemic inflammatory response index (SIRI) were independently associated with a lower SpO_2_. **Conclusions:** Our results suggest that albumin and specific haematological parameters are promising markers of reduced oxygen saturation in OSA.

## 1. Introduction

Obstructive sleep apnea (OSA) is a leading public health issue affecting over 900 million people worldwide [1,2]. In Italy, the most recent epidemiological data published by the Ministry of Health estimate that moderate-severe and mild-moderate OSA affect 27% and 54% of the adult population, respectively [3].

OSA is characterised by intermittent and repeated episodes of upper airway collapse during sleep, resulting in partial (hypopnea) or complete (apnea) airflow obstruction [4]. This causes excessive daytime sleepiness with frequent awakenings, characterised by repeated episodes of intermittent hypoxia followed by rapid reoxygenation [5]. Patients with OSA are known to have a high risk of developing ischemic heart disease, heart failure, arrhythmia, stroke, and transient ischemic attack [6,7].

The cycles of deoxygenation and re-oxygenation caused by intermittent hypoxemia in patients with OSA have been shown to induce an excessive production of reactive oxygen species (ROS), which causes structural and functional damage to proteins, DNA, and lipids [8,9]. In addition, intermittent hypoxemia is associated with the production of pro-inflammatory factors, metabolic dysregulation, and platelet aggregation [10].

Although the exact mechanisms involved are not fully understood, several studies suggest that, in OSA patients, oxidative stress and systemic inflammation play a key pathophysiological role in the onset and progression of cardiovascular morbidity and vascular dysfunction [11,12].

The gold standard for the diagnosis of OSA, polysomnography (PSG), allows the systematic collection of various parameters simultaneously during sleep. However, it requires access to specialised centres and equipment [13].

Therefore, several investigators have proposed the use of haematologically based inflammatory and oxidative stress parameters as easily measurable markers for the early diagnosis and monitoring of OSA [14,15]. Tumour necrosis factor (TNF), C-reactive protein (CRP), and interleukins-6 (IL-6) represent the most studied inflammatory markers in OSA, and their increase has been observed in OSA, patients compared to controls [16]. Additionally, the use of alternative hematological parameters present in the blood count as specific biochemical markers of the disease has recently gained increasing interest [15]. Such parameters, e.g., the neutrophil to lymphocyte ratio (NLR), platelet to lymphocyte ratio (PLR), and monocyte to lymphocyte ratio (MLR), have been investigated in several disease states [17,18,19,20,21]. A recent meta-analysis reported that the NLR could be a reliable marker to evaluate systemic inflammation and predict the severity of disease in OSA patients [22]. Among the markers of oxidative stress, albumin is the most abundant circulating protein that possesses significant antioxidant activity and is routinely measured [23]. A reduction of the antioxidant properties of serum albumin has been shown to be significantly associated with the presence and severity of OSA [24].

The apnea-hypopnea index (AHI), which indicates the number of apneas or hypopneas recorded per hour of sleep, is commonly used to diagnose OSA and determine its severity [25]. However, the AHI does not consider the duration of respiratory cessations and subsequent intermittent oxygen desaturations, which seem to play an important role in the development of complications related to OSA [26]. Recently, alternative parameters such as oxygen saturation (SpO_2_), percentage of time with SpO_2_ below 90%, and oxygen desaturation index (ODI), have also been used to assess the severity of OSA [27,28,29]. In this context, the aim of our study was to examine the relation between specific haematological parameters as markers of oxidative and inflammatory stress and the degree of hypoxia, measured by AHI, ODI, and SpO_2_, in a cohort of OSA patients in order to identify promising biomarkers of the disease.

## 2. Methods

### 2.1. Study Population

We conducted a retrospective study of a consecutive series of patients with OSA, diagnosed by polysomnography (PSG), attending the Respiratory Disease Unit of the University Hospital of Sassari, north Sardinia (Italy), between 2015 and 2019. Patients with AHI < 5 events/h, central sleep apnea, and receiving treatment were excluded.

The following parameters were collected in each patient: age, sex, body mass index (BMI), smoking status, comorbidities, pharmacological treatment, cholesterol and LDL cholesterol, hepatic profile, alanine amino-transferase (ALT), aspartate amino-transferase (AST), white blood cell count (WBC), monocytes, lymphocytes, neutrophils, platelets, haemoglobin distribution width (HDW), red cell distribution width (RDW), mean platelet volume (MPV), albumin. Moreover, combined indices of inflammation were derived: NLR, PLR, MLR, systemic inflammation index (SII: neutrophils × platelets/lymphocytes), systemic inflammatory response index (SIRI: neutrophils × monocytes/lymphocytes), and aggregated index of systemic inflammation (AISI: neutrophils × platelets × monocytes/lymphocytes) [15,30].

Finally, AHI, ODI, and SpO_2_ mean values during PSG were collected from each patient. Apnea events were defined as the absence, or almost total absence, of oro-nasal airflow for a period ≥ 10 s. Hypopnea was defined as respiratory events by an airflow reduction of ≤50% of baseline for ≥10 s [25]. The AHI indicates the average number of apnea and hypopnea episodes per hour of sleep, while the ODI measures the number of desaturation events per hour, where desaturation events represent a decrease in the mean oxygen saturation of ≥3% for at least 10 s. Patients were classified into 3 separate groups based on their AHI scores: mild (5 ≤ AHI < 15), moderate (15 ≤ AHI < 30), and severe OSA (AHI ≥ 30) [3].

### 2.2. Statistical Analysis

Data are expressed as means (mean ± SD) or medians (median and IQR). The variable distribution was assessed using the Kolmogorov–Smirnov test. Correlations between variables were assessed using Spearman’s or Pearson’s correlation. Multiple linear regression analysis was used to assess the presence of independent associations between AHI, ODI, and SpO_2_ mean and clinical (BMI, smoking status, comorbidities, pharmacological treatment), demographic (age, sex), and laboratory variables (total and LDL cholesterol, ALT, AST, WBC, monocytes, lymphocytes, neutrophils, platelets, HDW, RDW, MPV, albumin, NLR, PLR, MLR, SII, SIRI, and AISI) by correcting for confounders with a *p* < 0.05 in univariate analysis. Non-normally distributed variables were log10-transformed prior to analysis using parametric tests. To avoid collinearity bias, the independent association of neutrophils, monocytes, NLR, PLR, MLR, SII, SIRI, and AISI with AHI, ODI, and SpO_2_ was assessed in separate models. Statistical analyses were performed using MedCalc for Windows, version 20.109-64 bit (MedCalc Software, Ostend, Belgium).

## 3. Results

Our study population included 269 subjects with OSA. According to the AHI, 32 out of 259 patients (12.35%) had mild, 61 (23.5%) moderate, and 166 (64%) severe OSA. The demographic, clinical, and laboratory data of the study population are presented in Table 1. OSA patients were predominantly male (75%), with a mean age of 60.4 (±12.6) years. The majority were obese, with a median BMI of 33.2 kg/m^2^ (IR: 25.7 to 38.8), whereas 109 out of 259 were not smokers. Hypertension, dyslipidemia, and diabetes were present in 69.5%, 54.3%, and 24%, respectively.

In univariate correlation analysis (Table 2), a significant positive relationship was observed between BMI and AHI (r = 0.03, *p* = 0.003), and between BMI and ODI (r = 0.26, *p* = 0.0001), whereas a significant negative association was observed between BMI and SpO_2_ med (r = −0.4, *p* < 0.0001).

No significant correlation was observed between AHI, ODI, or SpO_2_ med and age, sex, smoking status, or comorbidities, with the only exception of dyslipidaemia, which was negatively correlated with AHI (r = −0.16, *p* = 0.011) and ODI (r = −0.13, *p* = 0.03). AHI was correlated with the use of statins (r = −0.13, *p* = 0.03), while metformin treatment was negatively associated with ODI (r = 0.14, *p* = 0.026) and positively associated with SpO_2_ med (r = −0.16, *p* = 0.0077).

Significant positive relationships were observed between AHI and ODI and the following laboratory parameters: HDW (AHI: r = 0.13, *p* = 0.028; ODI: r = 0.14, *p* = 0.02), RDW (AHI: r = 0.15, *p* = 0.017; ODI: r = 0.16, *p* = 0.001), WBC (AHI: r = 0.16, *p* = 0.008; ODI: r = 0.18, *p* = 0.004), and neutrophils (AHI: r = 0.2, *p* = 0.0013; ODI: r = 0.23, *p* = 0.0001).

SpO_2_ was associated positively with HDW (r = 0.21, *p* = 0.0007) and negatively with RDW (r = −0.25, *p* < 0.0001), WBC (r = −0.15, *p* = 0.01), neutrophils (AHI: r = −0.22, *p* = 0.0002), and monocytes (r = −0.14, *p* = 0.02).

Regarding the combined inflammation indices, the AHI correlated positively only with NLR (r = 0.12, *p* = 0.05) and SII (r = 0.115, *p* = 0.006). ODI was positively correlated with NLR (r = 0.16, *p* = 0.0075), SII (r = 0.16, *p* = 0.009), SIRI (r = 0.15, *p* = 0.013), and AISI (r = 0.15, *p* = 0.015). Moreover, SpO_2_ showed a negative and more significant relationship with the same indexes (NLR: r = 0.21, *p* =0.0002, SII: r = 0.21, *p* = 0.0006, SIRI: r = −0.24, *p* = 0.0001, AISI: r = −0.2, *p* = 0.0002) and with MLR (r = −0.15, *p* = 0.014). Finally, there was a significant positive relationship between albumin and SpO_2_ (r = 0.3, *p* < 0.0001), and a negative correlation between albumin and AHI (r = −0.14, *p* = 0.003) and ODI (r = −0.16, *p* = 0.013).

In multivariate regression analysis, the AHI was independently associated with the BMI in all the models studied (Table 3). The ODI was independently correlated with BMI, hypercholesterolemia, and metformin (Table 4). While no association was observed between AHI or ODI and inflammatory parameters, particularly NLR, SII, SIRI, and AISI, that SpO_2_ was strongly and independently associated with BMI, albumin, and specific inflammatory parameters, e.g., neutrophils, monocytes, and SIRI (Table 5).

## 4. Discussion

OSA is known to be associated with different cardiovascular, metabolic, and neurodegenerative disease states [31]. In this condition, oxidative stress and inflammation, induced by intermittent hypoxia, seem to play a key pathophysiological role in OSA [32]. Several blood cell components and combined cell count indexes are increasingly being studied as markers of inflammation in several inflammatory disorders, including OSA [17,19,32,33]. However, only a few reports have evaluated the prognostic capacity of these indexes, primarily NLR and PLR, with conflicting results [18,22]. The investigation of the systemic inflammatory response index (SII) in OSA was performed only in two studies reporting a significant positive correlation between AHI and SII [34,35].

We sought to address this issue by investigating promising haematological parameters associated with intermittent hypoxia in OSA patients, including the combined indices of inflammation (NLR, MLR, PLR, SII, SIRI, and AISI). In fact, it has been shown that these indices provide more information about the presence of an inflammatory state when compared with individual blood cell types [36]. Multivariate analysis showed no significant associations between measures of disease severity, AHI, and simple or combined indices, although NLR and SII were associated with AHI in univariate correlation analysis, in accordance with previous studies [34,35].

Considering the current interest in alternative parameters to assess the severity of OSA [25,26], we also investigated the association between the inflammatory parameters and the grade of hypoxia, using the ODI and oxygen saturation.

The ODI was not independently associated with any blood parameter. The SpO_2_ mean exhibited a significant negative correlation with neutrophil and monocyte counts and with all the combined indexes barring PLR (*p* = 0.94). In multivariate analysis, neutrophil and monocyte counts and SIRI were independently associated with SpO_2_ mean reduction.

Chronic intermittent hypoxia and sleep fragmentation interact variably with the immune system, which triggers pro-inflammatory pathways and cellular activation [37]. In this report, the cells most affected by oxygen saturation appear to be neutrophils and monocytes. The neutrophils possess the ability to produce ROS under hypoxic conditions with an increase in recruitment, stimulation of degranulation, and survival of neutrophils through several pathways, including the NF-κB binding of hypoxia-inducible factor (HIF−1), the main regulator of oxygen homeostasis [38,39]. Additionally, it has been observed that the induced hypoxic stress is associated with an increase in monocytes and with the activation of the production of inflammatory mediators by these cells [40].

In our study, the SIRI parameter, which combines neutrophils and monocytes in relation to lymphocytes, further confirmed the presence of a significant association between alterations in these cells and a reduction of the mean saturation.

We also demonstrated that mean SpO_2_ was strongly and independently associated with serum albumin in all models examined, whereas no correlation between AHI and ODI with albumin was found. Serum albumin is an important antioxidant agent, and structural changes caused by free radicals interfere with its antioxidant properties. In OSA, it has been reported that the reduction of antioxidant capacity and the consequent aggravation of oxidative stress can also contribute to cardiovascular and metabolic abnormalities [24]. Additionally, it has been observed that ischemia-modified albumin (IMA), created by the modification of albumin by free radicals, increased significantly in severe OSA patients, and its elevation seems to be reversed by CPAP treatment [41].

Our results showed that SpO_2_ can be mainly related to the reduction of antioxidant properties induced by intermittent hypoxia when compared with the index of severity of AHI. In this context, studies on the evaluation of the association between IMA and the degree of hypoxia could be useful to further confirm our data.

Finally, we confirmed data previously obtained in other studies, showing that the severity of OSA and oxygen saturation are modulated by weight changes [42,43]. In fact, a significant positive relationship between AHI, ODI, and BMI was observed, and a higher BMI strongly correlated with a lower SpO_2_ mean. These results further highlight that the presence of obesity results in more severe episodes of obstruction and desaturation events and, consequently, lower oxygen saturation during polysomnography [44].

Our study has some limitations, such as the retrospective design, the relatively small sample size, and the lack of comparison with control subjects.

Nevertheless, this is the first study evaluating the role of SIRI, AISI, and albumin in OSA patients in correlation with different PSG parameters, particularly oxygen saturation. Moreover, it could be a reference study for future case-control research, which could better clarify the utility of the parameters analysed in the OSA condition.

In conclusion, these results suggest that lower oxygen saturation strongly correlates with a reduction in the antioxidant properties of albumin and a pro-inflammatory profile characterised by an increased number of monocytes and neutrophils and a higher systemic inflammatory response index (SIRI). Pending the results of larger prospective studies including control subjects, these findings suggest a possible role for oxygen saturation in assessing the inflammation and oxidative stress burden in OSA.

## Figures and Tables

**Table 1:** The demographic, clinical, and laboratory characteristics of the studied OSA patients.

Parameters	OSA Patients N = 259
**Age**	
***mean* (*±SD*)**	60.4 (±12.6)
**Sex. *n* (%)**	
**Female**	64 (25%)
**Male**	195 (75%)
**BMI (kg/m^2^)**	
***median* (*IR*)**	33.2 (29.2–38.8)
**ODI**	
***median*** **(*IR*)**	37.7 (21.5–58.6)
**AHI.**	
***median* (*IR*)**	35.5 (21.5–55.1)
**SpO_2_ mean (%)**	
***median* (*IR*)**	91.2 (88.3–93)
**Smoking *n* (%)**	
**Current smokers**	56 (22)
**Ex smokers**	94 (36)
**No smokers**	109 (42)
**Hypertension *n* (%)**	64 (69.5)
**ARB *n* (%)**	31 (33.7)
**Beta-blockers *n* (%)**	18 (19.5)
**ACE inhibitors *n* (%)**	27 (29.4)
**Calcium channel blockers. *n* (%)**	11 (12)
**Diuretics. *n* (%)**	27 (29.3)
**Dyslipidemia *n* (%)**	50 (54.3)
**Statins *n* (%)**	41 (45.6)
**Diabete mellitus *n* (%)**	22 (24)
**Metformin *n* (%)**	16 (17.4)
**Insulin *n* (%)**	10 (11)
**MPV (fL).**	
***median*** **(*IR*)**	8.4 (7.9–9.2)
**HDW (g/dL)**	
***median*** **(*IR*)**	2.6 (2.4–2.9)
**RDW (%).**	
***median* (*IR*)**	13.5 (12.7–14.7)
**WBC (×10^3^/µL).**	
***median* (*IR*)**	7.6 (6.1–9.1)
**Neutrophil (×10^3^/µL)**	
***median* (*IR*)**	4.1 (3.2–5.5)
**Monocyte (×10^3^/µL)**	
***median* (*IR*)**	0.5 (0.4–0.7)
**Lymphocytes (×10^3^/µL)**	
***median* (*IR*)**	2.2 (1.8–2.8)
**Platelets (×10^3^/µL)**	
***median* (*IR*)**	225 (191–265)
**NLR**	
***Median* (*IR*)**	1.9 (1.4–2.7)
**PLR**	
***median* (*IR*)**	100.7 (80.2–126.2)
**MLR**	
***median* (*IR*)**	0.2 (0.2–0.3)
**SII.**	
***median* (*IR*)**	414.2 (296–606.5)
**SIRI**	
***median* (*IR*)**	8.3 (0.7–1.6)
**AISI**	
***median* (*IR*)**	221.4 (139–369.1)
**Albumin (g/dL)**	
***mean*** ** (*±SD*)**	3.8 (±0.31)
**Cholesterol (mg/dL)**	
***mean*** ** (*±SD*)**	171.4 (±41.5)
**LDL chol. (mg/dL)**	
***mean*** ** (*±SD*)**	108.3 (±34.5)
**ALT (U/L)**	
***median* (*IR*)**	20 (15–31)
**AST (U/L)**	
***median* (*IR*)**	18 (15–23)

OSA: obstructive sleep apnea; BMI: body mass index; ODI: oxygen desaturation index; AHI: apnea-hypopnea index; angiotensin II receptor antagonist, ARB; MPV: mean platelet volume; HDW: haemoglobin distribution width; RDW: red cell distribution width; WBC: white blood cells; NLR: neutrophils to lymphocyte ratio; PLR: platelet to lymphocyte ratio; MLR: monocyte to lymphocyte ratio; SII: systemic inflammation index; SIRI: systemic inflammatory response index; AISI: aggregated index of systemic inflammation; LDL: low-density lipoprotein; ALT: alanine amino-transferase; AST: aspartate amino-transferase, IR: interquartile range; SD: standart deviation.

**Table 2:** Correlations between PSG parameters and demographic, clinical, and laboratory characteristics of the studied OSA patients.

OSA Parameters N = 259	AHI		ODI		SpO_2_ Mean	
	*r*	*p-Value*	*r*	*p-Value*	*r*	*p-Value*
**BMI (kg/m^2^)**	0.003	0.003 *	0.26	0.0001 *	−0.40	<0.0001 *
**AGE**	−0.01	0.81	0.02	0.74	−0.12	0.06
**Sex**	0.04	0.51	0.06	0.3	−0.1	0.09
**Smoking status**	0.021	0.73	0.002	0.97	−0.11	0.06
**Hypertension**	0.02	0.7	0.014	0.82	−0.0368	0.55
**Angiotensin receptor blocker**	0.0013	0.98	0.017	0.78	−0.13	0.026 *
**Beta-blockers**	0.09	0.14	0.06	0.34	−0.01	0.80
**ACE inhibitors**	−0.03	0.66	−0.02	0.7	0.03	0.63
**Calcium channel blockers**	−0.03	0.6	−0.02	0.7	−0.03	0.63
**Diuretics**	0.02	0.72	0.017	0.78	−0.06	0.28
**Dyslipidaemia**	−0.16	0.011 *	−0.13	0.03 *	0.04	0.51
**Statins**	−0.131	0.03 *	−0.11	0.06	0.05	0.38
**Diabete mellitus**	0.05	0.43	0.09	0.11	−0.09	0.14
**Metformin**	0.06	0.3367	0.14	0.026 *	−0.16	0.0077 *
**Insulin**	0.04	0.53	0.07	0.25	−0.11	0.07
**MPV (fL)**	−0.04	0.46	−0.08	0.17	0.11	0.06
**HDW (g/dL)**	0.13	0.028 *	0.14	0.02 *	0.21	0.0007 *
**RDW (%)**	0.15	0.017 *	0.16	0.001 *	−0.25	<0.0001 *
**WBC (×10^3^/µL)**	0.16	0.008 *	0.18	0.004 *	−0.15	0.01 *
**Neutrophil (×10^3^/µL)**	0.2	0.0013 *	0.23	0.0001 *	−0.22	0.0002 *
**Monocyte (×10^3^/µL)**	0.05	0.4	0.06	0.32	−0.14	0.02 *
**Lymphocytes (×10^3^/µL)**	0.07	0.23	0.03	0.6	0.04	0.5
**Platelets (×10^3^/µL)**	0.05	0.35	0.07	0.22	−0.02	0.65
**NLR**	0.12	0.05 *	0.16	0.0075 *	−0.21	0.0002 *
**PLR**	−0.08	0.21	−0.04	0.52	0.004	0.94
**MLR**	−0.02	0.66	−0.005	0.94	−0.153	0.014 *
**SII**	0.115	0.006 *	0.16	0.009 *	−0.21	0.0006 *
**SIRI**	0.12	0.07	0.15	0.013 *	0.24	0.0001 *
**AISI**	0.1	0.08	0.15	0.015 *	−0.23	0.0002 *
**ALBUMIN (g/dL)**	−0.14	0.03 *	−0.16	0.013 *	0.3	<0.0001 *
**Cholesterol (mg/dL)**	−0.07	0.25	−0.08	0.18	0.12	0.07
**LDL chol (mg/dL)**	−0.03	0.6	−0.03	0.7	0.14	0.035 *
**ALT (U/L)**	−0.05	0.46	−0.02	0.67	−0.02	0.77
**AST (U/L)**	−0.02	0.8	−0.02	0.75	0.02	0.7

OSA: obstructive sleep apnea; BMI: body mass index; ODI: oxygen desaturation index; AHI: apnea-hypopnea index; angiotensin II receptor antagonist, ARB; MPV: mean platelet volume; HDW: haemoglobin distribution width; RDW: red cell distribution width; WBC: white blood cells; NLR: neutrophils to lymphocyte ratio; PLR: platelet to lymphocyte ratio; MLR: monocyte to lymphocyte ratio; SII: systemic inflammation index; SIRI: systemic inflammatory response index; AISI: aggregated index of systemic inflammation; LDL chol: low-density lipoprotein cholesterol; ALT: alanine amino-transferase; AST: aspartate amino-transferase. IR: interquartile range; SD: standart deviation; * Numbers in bold font indicate statistical significance.

**Table 3:** Correlation between AHI and demographic, clinical, and laboratory characteristics of the studied population obtained by multivariate regression analysis.

	r_partial_	*p-Value*
**BMI (kg/m^2^)**	0.2061	0.0028 *
**Hypercholesterolemia**	−0.0752	0.2802
**Statin**	−0.0086	0.9017
**HDW (g/dL)**	−0.0071	0.9179
**RDW (%)**	0.0643	0.3558
**Albumin (g/dL)**	−0.0591	0.3962
**WBC (×10^3^/µL)**	0.0578	0.4065
**BMI (kg/m^2^)**	0.2109	0.0022 *
**Hypercholesterolemia**	−0.0731	0.2941
**Statin**	−0.0109	0.8759
**HDW (g/dL)**	−0.0081	0.9070
**RDW (%)**	0.0662	0.3421
**Albumin (g/dL)**	−0.0555	0.4257
**Neutrophils (×10^3^/µL)**	0.0265	0.7036
**BMI (kg/m^2^)**	0.2246	0.0011 *
**Hypercholesterolemia**	−0.0740	0.2879
**Statin**	−0.0105	0.8798
**HDW (g/dL)**	−0.0094	0.8929
**RDW (%)**	0.0748	0.2826
**Albumin (g/dL)**	−0.0604	0.3861
**NLR**	−0.0220	0.7520
**BMI (kg/m^2^)**	0.2269	0.0010 *
**Hypercholesterolemia**	−0.0741	0.2869
**Statin**	−0.0102	0.8831
**HDW (g/dL)**	−0.0087	0.9001
**RDW (%)**	0.0728	0.2954
**Albumin (g/dL)**	−0.0584	0.4018
**SII**	−0.0120	0.8626

BMI: body mass index; HDW: haemoglobin distribution width; RDW: red cell distribution width; WBC: white blood cells; NLR: neutrophils to lymphocyte ratio; SII: systemic inflammation index; * Numbers in bold font indicate statistical significance.

**Table 4:** Correlation between ODI and demographic, clinical, and laboratory characteristics of the studied population obtained by multivariate regression analysis.

	r_partial_	*p-Value*
**BMI (kg/m^2^)**	0.2151	0.0018 *
**Hypercholesterolemia**	−0.1801	0.0092 *
**Metformin**	0.1877	0.0066 *
**HDW (g/dL)**	−0.0061	0.9300
**RDW (%)**	0.0576	0.4086
**Albumin (g/d** **L** **)**	−0.0811	0.2437
**WBC (×10^3^/µL)**	0.0783	0.2606
**BMI (kg/m^2^)**	0.2113	0.0022 *
**Hypercholesterolemia**	−0.1777	0.0102 *
**Metformin**	0.1886	0.0064 *
**HDW(g/dL)**	−0.0054	0.9380
**RDW (%)**	0.0534	0.4431
**Albumin (g/dL)**	−0.0735	0.2909
**Neutrophils (×10^3^/µL)**	0.0762	0.2737
**BMI (kg/m^2^)**	0.2458	0.0003 *
**Hypercholesterolemia**	−0.1820	0.0085 *
**Metformin**	0.1849	0.0075 *
**HDW (g/dL)**	−0.0103	0.8819
**RDW (%)**	0.0543	0.4353
**Albumin (g/dL)**	−0.0667	0.3380
**NLR**	0.0622	0.3714
**BMI (kg/m^2^)**	0.2384	0.0005 *
**Hypercholesterolemia**	−0.1850	0.0075 *
**Metformin**	0.1883	0.0064 *
**HDW (g/dL)**	−0.0155	0.8241
**RDW (%)**	0.0570	0.4132
**Albumin (g/dL)**	−0.0693	0.3199
**SII**	0.0774	0.2662
**BMI (kg/m^2^)**	0.2394	0.0005 *
**Hypercholesterolemia**	−0.1805	0.0091 *
**Metformin**	0.1867	0.0069 *
**HDW (g/dL)**	−0.0144	0.8359
**RDW (%)**	0.0606	0.3845
**Albumin (g/dL)**	−0.0727	0.2967
**SIRI**	0.0512	0.4621
**BMI (kg/m^2^)**	0.2301	0.0008 *
**Hypercholesterolemia**	−0.1837	0.0079 *
**Metformin**	0.1912	0.0057 *
**HDW (g/dL)**	−0.0212	0.7610
**RDW (%)**	0.0611	0.3801
**Albumin (g/dL)**	−0.0735	0.2914
**AISI**	0.0746	0.2842

BMI: body mass index; HDW: haemoglobin distribution width; RDW: red cell distribution width; WBC: white blood cells; NLR: neutrophils to lymphocyte ratio; SII: systemic inflammation index; SIRI: systemic inflammatory response index; AISI: aggregated index of systemic inflammation; * Numbers in bold font indicate statistical significance.

**Table 5:** Correlation between mean SpO_2_ and demographic, clinical, and laboratory characteristics of the studied population obtained by multivariate regression analysis.

	r_partial_	*p-Value*
**BMI (kg/m^2^)**	−0.3640	<0.0001 *
**ARB**	−0.0457	0.5387
**Metformin**	−0.1014	0.1722
**HDW (g/dL)**	−0.0066	0.9292
**RDW (%)**	−0.0305	0.6815
**Albumin (g/dL)**	0.2219	0.0025 *
**LDL Chol (mg/dL)**	0.0313	0.6732
**WBC (×10^3^/µL)**	−0.1353	0.0679
**BMI (kg/m^2^)**	−0.3516	<0.0001 *
**ARB**	−0.0379	0.6105
**Metformin**	−0.1076	0.1471
**HDW (g/dL)**	−0.0115	0.8767
**RDW (%)**	−0.0162	0.8275
**Albumin (g/dL)**	0.2101	0.0043 *
**LDL Chol (** **mg/dL)**	0.0328	0.6589
**Neutrophils (×10^3^/µL)**	−0.1517	0.0404 *
**BMI (kg/m^2^)**	−0.3619	<0.0001 *
**ARB**	−0.0626	0.3992
**Metformin**	−0.1236	0.0956
**HDW (g/dL)**	0.0072	0.9225
**RDW (%)**	−0.0445	0.5494
**Albumin (g/dL)**	0.2228	0.0024 *
**LDL Chol (mg/dL)**	0.0208	0.7794
**Monocytes (×10^3^/µL)**	−0.2030	0.0058 *
**BMI (kg/m^2^)**	−0.3960	<0.0001 *
**ARB**	−0.0249	0.7378
**Metformin**	−0.1002	0.1773
**HDW (g/dL)**	−0.0024	0.9742
**RDW (%)**	−0.0348	0.6395
**Albumin (g/dL)**	0.2070	0.0049 *
**LDL Chol (mg/dL)**	0.0173	0.8158
**NLR**	−0.0671	0.3668
**BMI (kg/m^2^)**	−0.3975	<0.0001 *
**ARB**	−0.0308	0.6787
**Metformin**	−0.1071	0.1489
**HDW (g/dL)**	0.0066	0.9286
**RDW (%)**	−0.0492	0.5080
**Albumin (g/dL)**	0.2071	0.0049 *
**LDL Chol (mg/dL)**	0.0083	0.9110
**MLR**	−0.0808	0.2764
**BMI (kg/m^2^)**	−0.3921	<0.0001 *
**ARB**	−0.0372	0.6166
**Metformin**	−0.0983	0.1852
**HDW (g/dL)**	−0.0002	0.9976
**RDW (%)**	−0.0508	0.4943
**Albumin (g/dL)**	0.2148	0.0035 *
**LDL Chol (mg/dL)**	0.0170	0.8189
**SII**	−0.0052	0.9442
**BMI (kg/m^2^)**	−0.3951	<0.0001 *
**ARB**	−0.0192	0.7963
**Metformin**	−0.1186	0.1099
**HDW (g/dL)**	0.0072	0.9228
**RDW (%)**	−0.0179	0.8091
**Albumin (g/dL)**	0.2088	0.0046 *
**LDL Chol (mg/dL)**	0.0184	0.8047
**SIRI**	−0.1935	0.0087 *
**BMI (kg/m^2^)**	−0.3828	<0.0001 *
**ARB**	−0.0336	0.6516
**Metformin**	−0.1063	0.1522
**HDW (g/dL)**	0.0027	0.9710
**RDW (%)**	−0.0340	0.6475
**Albumin (g/dL)**	0.2121	0.0039 *
**LDL Chol (mg/dL)**	0.0291	0.6951
**AISI**	−0.1132	0.1272

BMI: body mass index; ARB: angiotensin II receptor antagonist; HDW: haemoglobin distribution width; RDW: red cell distribution width; LDL chol: low-density lipoprotein cholesterol; WBC: white blood cells; NLR: neutrophils to lymphocyte ratio; MLR: monocyte to lymphocyte ratio; SII: systemic inflammation index; SIRI: systemic inflammatory response index; AISI: aggregated index of systemic inflammation. * Numbers in bold font indicate statistical significance.

## Data Availability

All data relevant to the study are included in the article.
